# Cellulose/Polyhydroxybutyrate (PHB) Composites as a Sustainable Bio-Based Feedstock to 3D-Printing Applications

**DOI:** 10.3390/ma17040916

**Published:** 2024-02-16

**Authors:** Lucia D’Arienzo, Stefano Acierno, Antonella Patti, Luciano Di Maio

**Affiliations:** 1 Department of Industrial Engineering, University of Salerno, Via Giovanni Paolo II 132, 84084 Fisciano, Italy; ldarienzo@unisa.it (L.D.); ldimaio@unisa.it (L.D.M.); 2 Department of Engineering, University of Sannio, Piazza Roma 21, 82100 Benevento, Italy; 3 Department of Civil Engineering and Architecture (DICAr), University of Catania, Viale Andrea Doria 6, 95125 Catania, Italy; antonella.patti@unict.it

**Keywords:** 3D-printing, polyhydroxybutyrate (PHB), cellulose, thermal analysis, mechanical performance

## Abstract

In this work, we have studied the potential application for 3D-printing of a polymer made from combining a biodegradable and biocompatible polymer (i.e., polyhydroxybutyrate, PHB) with natural bio-based fiber (i.e., cellulose). To this end, a masterbatch at 15 wt.% in filler content was prepared by melt-blending, and then this system was “diluted” with pure PHB in a second extrusion phase in order to produce filaments at 1.5 and 3 wt.% of cellulose. For comparison, a filament made of 100% virgin PHB pellets was prepared under the same conditions. All the systems were then processed in the 3D-printer apparatus, and specimens were mainly characterized by static (tensile and flexural testing) and dynamic mechanical analysis. Thermogravimetric analysis, differential scanning calorimetry, spectroscopic measurements, and morphological aspects of PHB polymer and composites were also discussed. The results showed a significant negative impact of the process on the mechanical properties of the basic PHB with a reduction in both tensile and flexural mechanical properties. The PHB–cellulose composites showed a good dispersion filler in the matrix but a poor interfacial adhesion between the two phases. Furthermore, the cellulose had no effect on the melting behavior and the crystallinity of the polymer. The addition of cellulose improved the thermal stability of the polymer and minimized the negative impact of extrusion. The mechanical performance of the composites was found to be higher compared to the corresponding (processed) polymer.

## 1. Introduction

Plastics are among the most essential materials in daily life and industrial contexts due to their exceptional properties such as light weight, low maintenance requirements, weathering resistance, transparency, and low price [1]. Global thermoplastics production is predicted to reach one million metric tons in the next few years. Conventional plastics (polyethylene, polypropylene, polystyrene, polyvinylchloride, polyethylene terephthalate and polyurethane) break down in nature in around 20–100 years, representing a severe environmental problem for waste accumulation in the ecosystem [2]. Plastics may decompose into micro- and nano-plastics, posing a direct or indirect risk to human health through inhalation or digestion [3]. On the other side, plastic waste incineration can release various pollutants into the atmosphere, such as dioxins, furans, mercury, polychlorinated biphenyls nitrogen and carbon oxide, volatile organic compounds, ash, and residue [4].

Everyone is becoming more aware of the environmental impact of plastic products. Bioplastics, which are plastics partially or totally manufactured from bio-based components and renewable sources, are being proposed as a possible alternative to conventional petroleum-based polymers. Biopolymers can be made from polysaccharides, proteins, and lipids derived from plants or animals, bacterial activity, or a traditional synthesis of bio-sourced or synthetic monomers. Recently, biopolymers have found applications in product packaging [5], automobile and aviation engineering [6], tissue engineering [7], medical implant [8], water treatment [9], and geotechnical engineering [10].

Polyhydroxybutyrate (PHB) is the most prevalent form of polyhydroxyalkanoates (PHAs) polymer, belonging to the polyester class. It is biodegradable and biocompatible, and it was generated as an internal energy storage material by numerous bacteria species, such as Alcaligenis euterophus, Bacillus, and Pseudomonas [11]. Under adverse conditions, such as nutrient shortage, PHB can be depolymerized and used as a food source by microbes [12]. PHB can be made from a variety of feedstocks, including glycerol, dairy waste, agro-industrial waste, food industry waste, and sugars [2]. PHB can be potentially applied in the development of implanted medical devices or in current pharmacology for the creation of novel drug delivery systems [12]. Combining PHB with other biodegradable polymers and fillers has become a popular strategy to improve its properties and biodegradability while cutting production costs. Wei et al. [13] investigated the thermophysical properties and biodegradation behavior of green systems made from PHB and potato peel waste. When compared to pure PHB, the biocomposites possessed lower mechanical performance but an exceedingly high biodegradation rate. These biocomposites can open up new possibilities for biomaterials that degrade quickly in agricultural and horticultural applications. Micro-sized products made entirely of lignin–cellulosic components extracted from olive mill wastewater (from the production of olive oil) were incorporated in a PHB matrix to develop natural films, for agriculture and food packaging, through the solvent-cast technique [14]. Bioplastic-based films displayed several benefits such as low cost, renewable nature, aqueous and soil quick degradability as well as significant drawbacks such as low mechanical resistance and complicated manufacturing processes. Almond shells, rice husks and seagrass were investigated as lignocellulosic wastes fillers in PHB/fiber composites for food packaging applications [15]. All of the fillers were demonstrated to be suitable for matrix reinforcement without altering the crystallinity or degradation rate of PHB.

In the last 30 years, additive manufacturing (AM) has evolved as a new digital technology that is versatile, flexible, and highly adjustable for creating three-dimensional items with complicated geometry and precise shape using computer-aided design (CAD) [16]. Metallic, ceramic, and polymeric materials, as well as mixtures in the form of composites and hybrid systems, can be used to produce 3D parts/objects [17,18,19]. Given the wide range of material flexibility and design freedom, efforts have been focused on producing bio-based filaments to develop sustainable 3D-printed objects and replace fossil-based conventional plastic filaments [20,21]. One of the key issues in the FDM process is the use of innovative, sustainable biomaterials. However, PHB’s low thermal stability and its tendency to decompose set considerable limits to melt processing. Strategies to improve the printability of PHB polymer and the final properties of 3D-printed parts comprehend the use of copolymers such as poly(3-hydroxybutyrate-*co*-3-hydroxyhexanoate) (PHBH) [22] and poly(3-hydroxybutyrate-*co*-hydroxyvalerate) (PHBV) [23] or the blending with polylactide acid (PLA) [24], polycaprolactone (PCL) [25], or the combinations with particles such as zinc oxide (ZnO) [26], hydrous zirconium oxide (ZrO_2_·nH_2_O) [27], Kaolin [28], lignocellulosic corncob powder [29], and so on.

In this study, melt blending extrusion was utilized to make bio-based filaments from PHB polymers for use in fused deposition modeling (FDM), which is one of the most common additive techniques. As a natural filler, cellulose was chosen for different aspects: it is the fundamental structural constituent of the plant cell wall; it is the most used green reinforcement for composites; and it possesses outstanding biodegradability, non-abrasiveness, and low density [20]. Previous research has focused on PHB and lignocellulosic particles in 3D-printing applications, adding fillers and creating filaments using various procedures: (i) a unique extrusion step in a twin-screw extruder [30]; (ii) compounding the components in a mixer, and then passing through a co-rotating extruder [31]; (iii) solution mixing to combine polymer and cellulose nanocrystals, a second compounding in a high shear mixing, and extrusion in a capillary rheometer to create a filament [22]. Here, two extrusion phases have been provided to optimize the filler dispersion in the matrix and to generate a filament suited for the printer. Starting with the base components (PHB and cellulose powder), a system with 15% filler content (masterbatch) was produced, which was subsequently diluted in a second extrusion using pure polymer. Finally, a desktop filament extruder was used to prepare smooth filaments of appropriate size for use in the printing apparatus. The 3D-printed specimens were mainly characterized by static and dynamic mechanical properties. Differential scanning calorimetry (DSC), thermogravimetric analysis (TGA) (in isothermal conditions both in nitrogen and air atmosphere, and non-isothermal conditions), and spectroscopic investigations in attenuated total reflectance (ATR) were also performed on a masterbatch and pure polymer. Morphological aspects were finally discussed through the auxilium of microscopic analysis. It is well known that PHB decays quickly during extrusion, and the molecular weight drops following melt processing, resulting in filaments that are excessively brittle [32]. A characterization of virgin pellets treated twice in the extruder was also performed to better understand the effect of the manufacturing process and cellulose addition on the final performance of the basic polymer.

## 2. Materials and Methods

### 2.1. Materials

The poly(3-hydroxybutyrate) (cod. ENMAT PHB resin Y3000P) was supplied by TianAN Biopolymer (Ningbo, Zhejiang, China). As filler, ultra-fine and highly pure microcrystalline cellulose (MCC) powder (ARBOCEL^®^ UFC 100, purity of 99.5%, average particle size around 6–12 µm, bulk density of 0.15–0.2 g/cm^3^) was purchased from J. Rettenmaier & Söhne GmbH & Co. KG (Rosenberg, Baden-Wurttemberg, Germany).

### 2.2. Composites Preparation

Prior to any operation, all the ingredients were dried for 12 h in a vacuum oven at 70 °C to prevent hydrolysis and material degradation. Initially, a masterbatch (MB) at 15 wt.% of cellulose was prepared with a two-step procedure. The cellulose powder was manually mixed with the PHB granules to obtain a dispersion of the powder into the polymer. Then, the so-prepared cellulose–PHB system was processed in a co-rotating double-screw extruder (mod. Collin ZK25, L/D = 42, screw diameter of 25 mm) at 150 RPM with a temperature profile, from the hopper to the die, of 155 °C–160 °C–160 °C–160 °C–160 °C–160 °C–160 °C–150 °C.

The masterbatch was used to realize filaments with cellulose concentrations of 1.5% (PHB/MCC 1.5%) and 3% (PHB/MCC 3%). The two mixtures were prepared by mixing the pure polymer and the masterbatch with proportions of 90/10 and 80/20, respectively. To evaluate the effect of processing on the basic polymer, the poly(3-hydroxybutyrate) pellets were extruded two (PHB_2) times under the same conditions used for the composite preparation.

A desktop filament extruder (Precision 350 filament maker, 3DEVO, The Netherlands) was used to prepare filaments with a diameter of 2.85 mm. Each system was processed with a screw speed of 2.5 RPM and a distinct pre-optimized temperature profile. In particular, the hopper temperature was around 175 °C for virgin PHB pellets and composites with cellulose amounts lower than 1.5 wt.%. Systems with cellulose concentrations higher than 1.5 wt.% were more viscous and required an increase in hopper temperatures (180 °C).

**Figure 1:**
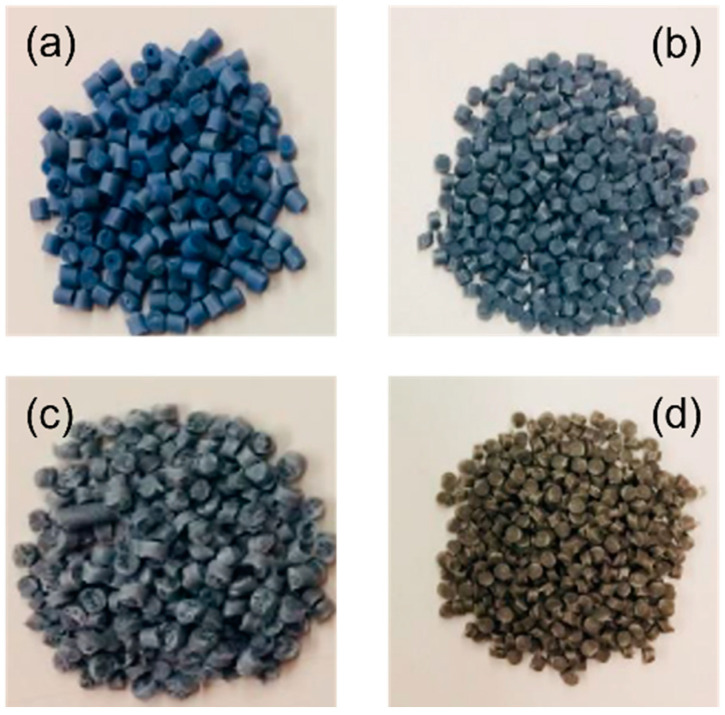
Photographs of: (**a**) virgin PHB pellets; (**b**) virgin PHB pellets after extrusion; (**c**) masterbatch; (**d**) masterbatch after extrusion.

The die temperature was set at 178 °C for the virgin PHB polymer and a few degrees lower for composites. In such systems, the PHB polymer partly decomposed and the viscosity decreased, which was verified through torque measurements during compounding in [33]. In these conditions, die temperature was reduced to facilitate material collection. Changes in color (darkening) was the first indication of the degradative phenomena that occurred in composites (Figure 1).

### 2.3. Three-Dimensional (3D) Printing

The filaments were processed in a desktop 3D-printer (3D Ultimaker, by Ultimaker, Utrecht, The Netherlands). Samples suitable for the mechanical characterization were printed by setting the support platform at ambient temperature, a layer thickness of 0.1 mm, a raster angle of 45°, a filling degree of 100%, and nozzle temperature of 185 °C. Different types of specimens were realized based on mechanical analysis: (i) a dog-bone shape for static tensile testing, 1.9 × 13 × 165 mm in size; (ii) a rectangular shape for static bending tests, 3.2 × 12.7 × 127 mm in size; and (iii) a rectangular shape for dynamic-mechanical analysis 2.5 × 12 × 60 mm in size.

### 2.4. Characterization Techniques

A thermogravimetric analyzer (TGA) measures changes in a sample’s weight as a function of temperature and time. This technique is often regarded as the most reliable for studying the thermal stability of polymeric materials, i.e., the temperature at which degradation processes occur. Measurements were carried out in a thermogravimetric analyzer (TGA-50, by Shimadzu, Kyoto, Japan) on virgin PHB and a masterbatch using samples with a mass of 10–20 mg. Under non-isothermal conditions, the samples were heated, at a rate of 10 °C/min, from ambient temperature up to 700 °C in nitrogen atmosphere with a flow rate of 20 mL/min. Under isothermal conditions, the samples were kept at a constant temperature, of 180 or 200 °C, for one hour in both air and nitrogen atmosphere.

Differential scanning calorimetry (DSC) measures enthalpy changes in materials due to changes in physical or chemical properties. Using a DSC apparatus (DSC822, Mettler Toledo, Ohio, USA), the samples were subjected to the following thermal cycle: (i) heating from 25 to 220 °C with a rate of 10 °C/min (1st Heating); (ii) isotherm at 220 °C for 5 min; (iii) cooling from 220 to 25 °C at a rate of 10 °C/min (Cooling); (iv) heating from 25 to 220 °C with a rate of 10 °C/min (2nd Heating). The degree of crystallinity ($X_{c}$) was calculated as:

(1) $$X_{c}=\frac{\Delta H_{f}}{\Delta H_{f}^{0}\;\left(1-\emptyset \right)}\cdot 100$$

where $\Delta H_{f}$ is the measured melting enthalpy in (J/g); $\Delta H_{f}^{0}$ is the melting enthalpy of pure PHB equal to 146 J/g [34] and ∅ is the cellulose content.

Morphological observations were performed using a field emission scanning electron microscope (FESEM, model LEO 1525), by Carl Zeiss GmbH (Oberkochen, Germany). The samples were cryogenically fragmented in liquid nitrogen and gold-sputtered in an Agar Auto Sputter Coater (mod. 108 A, Stansted, UK) at 30 mA for 160 s.

Spectroscopic analyses were performed using a Thermo Nicolet NEXUS 600 spectrophotometer with a Smart Performer accessory for the Attenuated Total Reflection mode (ATR/FT-IR). For each spectrum, 64 cyclical scans, with a resolution of 4 cm^−1^ and an acquisition range of 4000–650 cm^−1^, were performed.

The tensile and flexural testing of printed materials were performed in static mode on a dynamometer SANS CMT 6000, manufactured by MTS Systems (Eden Prairie, MN, USA), by using a load cell of 5 KN and speed of 5 mm/min, according to standards ASTM D638 and ASTM D790, respectively.

Dynamic mechanical analysis (DMA) was conducted using a DMA Q800 V7.5 Build 127 (TA Instrument, New Castle, DE, USA). The printed specimens were characterized in dual cantilever mode at a frequency of 1 Hz at 0.2% constant strain. The applied thermal program consisted of an initial cooling of the sample to −20 °C, an isothermal phase of 1 min at −20 °C, and a heating to 100 °C at a rate of 3 °C/min.

## 3. Results

### 3.1. Thermal and Morphological Aspects of Neat and Masterbatch PHB-Based Systems

**Figure 2:**
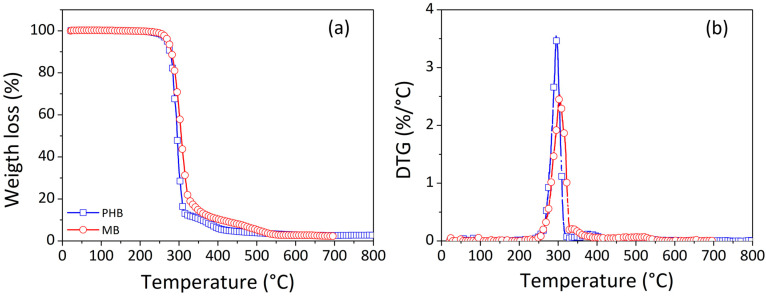
Weight loss percentage (%) (**a**) and DTG (%/°C) (**b**) as function of temperature (°C) for virgin PHB and masterbatch (MB). (Legend in Figure 2b as in (**a**).)

Figure 2 displays a comparison between the virgin polymer (PHB) and the masterbatch MB (i.e., the composite at 15 wt.% of filler loading) in terms of percentage of weight loss and its first derivative (DTG) as a function of temperature.

For both PHB and masterbatch, the thermogravimetric curves reveal that weight loss occurs with a two-step mechanism. The first degradation step takes place at temperatures around 250–300 °C, while the second occurs at temperatures in the range of 300–400 °C. The literature reports that the degradation process for PHB comprises chain scission and hydrolysis, and it results in a decrease in molecular weight; the thermal degradation of pure PHB proceeds abruptly with a single weight loss step [35,36] that starts at about 220 °C and ends at around 315 °C, leaving a weight residue of 2.32% [37]. As a consequence, the two-step dynamics observed for our sample can be attributed to the decomposition of some additives included in the PHB by the manufacturer (this hypothesis is confirmed also by the morphological analysis).

From a comparison between the two degradation curves (blue line for PHB and red lines for MB), it can be observed that the addition of cellulose improves the thermal stability of the PHB. Indeed, the results show a shift toward higher temperatures of the thermogram and in particular a sensible shift toward higher temperature of the peak of the DTG curve (i.e., the temperature at which the degradation rate is at its maximum).

Temperatures at specific weight loss percentages of 25%, 50%, 75% and the maximum degradation temperature (T_max_), calculated as the temperature at the peak of the DTG curve, for PHB and masterbatch, are summarized in Table 1. These data show that the MB samples reach the same weight loss percentage at higher temperatures compared to the pure matrix.

**Figure 3:**
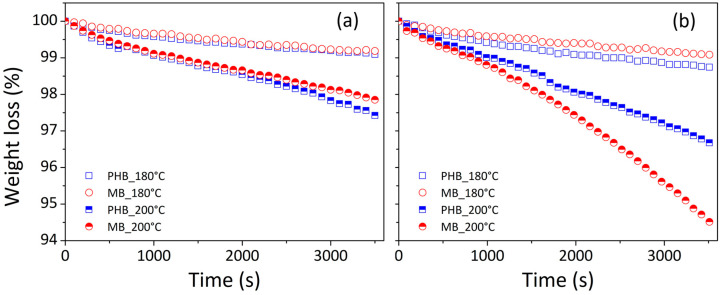
Weight loss percentage (%) vs. time (s) during thermogravimetric analysis performed in isothermal mode at 180 °C and 200 °C by using an atmosphere of nitrogen (**a**) or air (**b**) for virgin polymer (PHB) and masterbatch (MB).

Data (in Figure 3) show that the weight loss rates for the PHB and the composite at 15 wt.% of cellulose (MB) are strongly affected by the temperature both in nitrogen (Figure 3a) and air (Figure 3b) atmosphere: the higher the temperature, the greater the decomposition rate. In air (Figure 3b), at temperature of 180°, the degradation rates of the two materials are quite similar, whereas at 200 °C, the masterbatch decomposes faster than the PHB. In more detail, at 200 °C in air, the two curves became significantly distinct only after more than 1000 s.

Similar behaviors were observed in the case of ternary compounds made from PLA/PHB and cellulose nanocrystals [38]. At 200 °C, isothermal thermogravimetric analysis revealed that nanocrystal-based PLA/PHB blends had lower thermal stability than neat PLA/PHB. Dynamic–thermal measurements also revealed that adding cellulose to formulations shifted the onset of the PLA/PHB degradation process from 266 °C to around 278 °C.

The thermal behavior of cellulose/PHB composites can be correlated to different opposite mechanisms. Firstly, PHB thermal degradation almost exclusively involves a random chain scission (cis-elimination) reaction of the ester groups that forms oligomers and crotonic acid [39]. The crotonic acid can operate as an acid catalyst, resulting in the in situ hydrolysis of cellulose components and a significant drop in the fiber aspect ratio [40]. On the other side, the presence of crotonic acid seems also to induce a higher order in cellulose causing the disruption of some hydrogen bonds and promoting more stable cellulose structures [40]. Furthermore, the degradation of hemicellulose produces acetic acid, which promotes the random scission of ester linkages and thus the PHB deterioration [40].

Under nitrogen, and especially at low temperatures (180 °C), PHB and cellulose breakdown could be considered sluggish, and crotonic or acetic acid production could have no significant impact on compound deterioration. High temperature (200 °C) and oxidative environment could favor PHB and cellulose fiber deterioration, particularly over lengthy testing times (greater than 1000 s), reducing the thermal stability of the final products.

**Figure 4:**
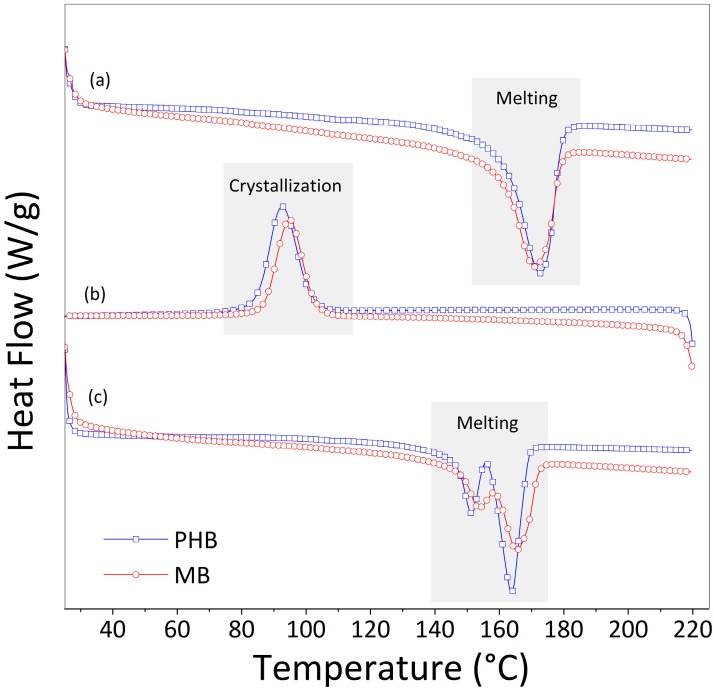
DSC thermograms of PHB and MB systems recorded during the thermal cycle, which began with a 1° sample heating (**a**), followed by cooling (**b**) and final 2° heating (**c**).

The DSC thermograms for the neat polymer and the masterbatch collected during the first heating, the cooling, and the second heating are reported in Figure 4.

Both the PHB polymer and composite at 15 wt.% of cellulose content show similar thermal events: an endothermic peak, corresponding to a melting process, during the first heating; an exothermic peak, corresponding to a crystallization process, during the cooling step; and a double peak, corresponding to melting processes, during the second heating. The single melting peak was recorded at about 172 °C during the first heating, whereas the double melting peak was recorded around 150 °C and 160 °C during the second heating. Multiple melting peaks are common in polyesters such as PHB [41] and related composites [42,43] and can be due to a variety of events, including recrystallization during the melting [44], changing of crystal perfection degrees [45], and the development of different crystalline forms [46]. For example, the melting process can involve melting some of the initial crystals as well as those formed during the melt-re-crystallization process during a heating scan. The lower endothermic peak could indicate the melting of the most original, less perfect, lamellae, whereas the higher temperature peak could correspond to the melting of thicker and more perfect lamellae generated through the recrystallization of partially melted material [45].

Furthermore, it should be taken into account that the PHB polymer has a low crystallization rate, and the cooling–heating rate used in DSC testing is probably too high to allow a complete crystallization [43]. During rapid cooling from a melt, reorganization and recrystallization can result in the creation of crystallites with varying thickness, distribution, stability, and perfection. Double endothermic behavior may result from the melting of lamellae with a bimodal thickness distribution or from the formation of different morphologies (such as spherulitic and crystal-aggregate structures), from different crystal structures (polymorphism) or perfect and imperfect crystals [41,45].

Another explanation of the lower temperature peak could be attributed to PHB degradation during the thermal cycle. The heating–cooling–heating program may lead to chain scission and the production of various molecular weight species, causing multiple melting behaviors [43].

Analysis of DSC data reveals no significant differences due to the presence of cellulose between the investigated materials: the degree of crystallinity remains constant at about 51–52%, and both melting (during first and second heating) and crystallization temperatures show negligible variations. DSC data, in terms of melting and crystallization temperatures, enthalpies, and crystallinity degree for each thermal stage (heating-cooling, heating), are reported in Table 2.

**Figure 5:**
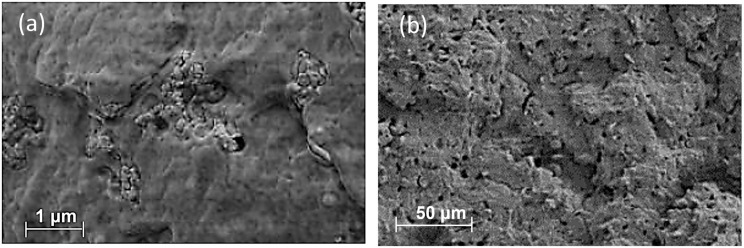
SEM micrographs of: (**a**) pure PHB polymer (30.00 KX), (**b**) cellulose-based composites at 15 wt.% in filler loading (MB) (1.00 KX).

SEM micrographs of pure polymer and composites containing 15 wt.% of cellulose are reported in Figure 5. The surface of the pristine polymer (see Figure 5a) shows the presence of a second phase in the form of (nano)spheres which probably correspond to some additive already speculated from the TGA analysis.

In the composite (see Figure 5b), the cellulose appears to be evenly distributed in the polymeric matrix. The good filler dispersion can be attributed to the intensive mixing and the prolonged processing time required for the filament preparation. Some holes and pores are visible on the sample surfaces; these cavities can be the result of debonding and fibril pull-out events that happened during the brittle fracture of cryogenically treated specimens, and these can be interpreted as an indicator of poor adhesion and compatibility between the two phases [47].

**Figure 6:**
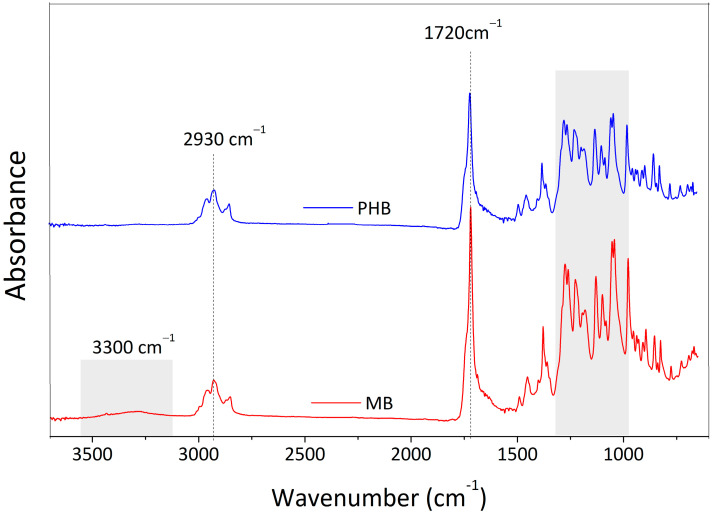
Normalized absorbance intensity in the wavenumber range of 4000–650 cm^−1^ for PHB and masterbatch system.

The ATR spectra of the virgin PHB and the composites at 15 wt.% of cellulose content, normalized to the intensity of the band at 2930 cm^−1^ (corresponded to the group CH_2_ that is present in all samples [48]), are compared in Figure 6.

According to previous literature [48], the inclusion of cellulose-based particles did not appreciably affect PHB characteristic bands. The spectral peaks of C-H stretching vibrations (methyl (CH_3_) and methylene (CH_2_)) were found between 2800 and 3000 cm^−1^, the peak that matched to the ester’s carbonyl (C=O) stretching was at 1720 cm^−1^, and the bands attributed to the C-H group bending and ester group stretching (C-O) were between 1000 and 1300 cm^−1^ [48,49]. A weak spectral band around 3300 cm^−1^ in the MB sample was linked to the presence of hydroxyl groups (O-H) in the cellulose component. These results can be regarded as experimental evidence that the two phases (matrix and filler) in the composites did not interact. Changes in the magnitude of the peaks could be attributed to degradation effects [50].

### 3.2. Mechanical Performance of 3D Parts Made from PHB/Cellulose Composites

#### 3.2.1. Static Mechanical Analysis

**Figure 7:**
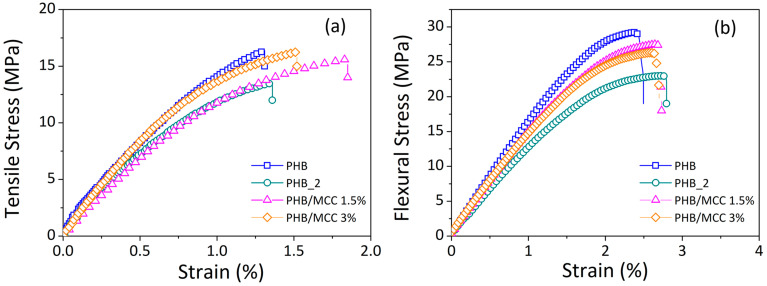
Representative stress–strain curves for 3D-printed samples made from basic PHB, processed PHB (PHB_2), and composites with 1.5 and 3% cellulose content, recorded during tensile testing (**a**) and flexural testing (**b**).

Representative stress–strain curves collected during the tensile (see Figure 7a) and flexural testing (see Figure 7b) of 3D specimens are reported in Figure 7, while a summary of the average tensile and flexural properties is reported in Table 3.

If the flexural and tensile behavior of the basic polymer (PHB) are compared to those of the polymer processed twice (PHB_2), a strong reduction in the stiffness is observed. This reduction in the mechanical properties of the polymer has to be attributed to a degradation caused by the extrusion process. Most industrial thermoplastic polymers are treated at temperatures over the melting point or glass transition (ranging from 400 to 650 K) in high-productivity processes such as extrusion, calendaring, and injection molding. Viscosities are expected to be 10–1000 times lower under these conditions, addressing various major technological and economic restrictions [51]. High pressures are generally used to allow the polymer to flow quickly through channels, dies, or molds. As a result, during typical polymer manufacturing, materials are subjected to thermal and mechanical stresses. Under these extreme circumstances, polymers become unstable and subject to non-structural irregularities, chain branching, and chain scission, all of which damage the macromolecular structural integrity [52]. The end result is a shift in molecular weight and/or physical characteristics [51,52]. The thermal decomposition of PHB is considered to be entirely caused by a non-radical random chain scission process [39]. PHB degrades at temperatures close to its melting point, and due to the narrow processing window, polymer chains may degrade during the operations [53].

The addition of cellulose to PHB reinforces both the tensile and the flexural response of the processed polymer (PHB_2) in different manners. The tensile modulus (E_t_) seems to increase as a function of the cellulose content; systems containing cellulose at 1.5% have almost the same modulus as the basic polymer (1680 vs. 1650 MPa), while the systems with 3% cellulose content slightly surpass the basic PHB (1720 vs. 1650 MPa). Under flexural conditions, small amounts of filler seem to improve the modulus of processed PHB (PHB_2), while higher amounts appear to determine scarce or negative effects. Indeed, an addition of 1.5 wt.% of cellulose seems to determine an increase in the flexural modulus with respect to the processed polymer (1520 vs. 1410 MPa), while the system containing 3 wt.% of cellulose shows almost the same modulus (1400 vs. 1410 MPa) and the same tensile strength (25 vs. 25 MPa) of the PHB_2. This behavior could be considered consistent with the observations of Chen et al. [54] who found that when the cellulose concentration surpasses a certain level, it does not provide further improvements in the mechanical properties of the composites. Indeed, they observed that the introduction of 1% cellulose increased the tensile strength of PHB from 27.5 to 33.3 MPa as well as the impact strength by 15%. Meanwhile, higher cellulose levels did not result in additional improvements of the mechanical properties. The authors attributed this behavior to three main factors: (1) the decreased crystallinity of the polymer due to the presence of cellulose can reduce tensile strength; (2) the higher cellulose strength and modulus compared to basic polymers support a reinforcement of tensile properties; (3) poor interfacial adhesion of two phases acts against reinforcing effects of the filler. The balance among these aspects determines the final properties of the system.

#### 3.2.2. Dynamic Mechanical Analysis (DMA)

**Figure 8:**
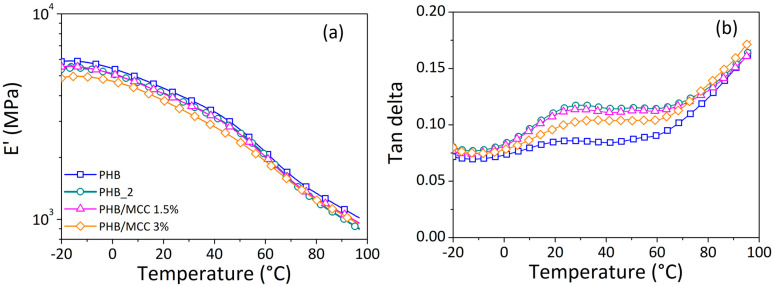
E’ in MPa (**a**) and tan delta (**b**) as a function of temperature (°C) for 3D-printed specimens made from basic PHB, processed PHB (PHB_2), and composites with 1.5 and 3% cellulose content.

The thermomechanical properties of the basic polymer (PHB), polymer processed twice (PHB_2), and composite at 1.5 or 3 wt.% cellulose loadings are displayed in terms of storage modulus (E’) and dissipation factor (tan delta) as a function of temperature in Figure 8a and Figure 8b, respectively.

The storage modulus decreases with increasing temperature, reflecting faster the relaxation processes of the material and the increased mobility of macromolecules at higher temperatures. The E’ curves for the composites are almost superimposed over that of PHB and PHB_2, showing at most a difference of about 10%, which is considered within the range of experimental errors. This finding could be considered a sign of negligible reinforcing effects of the filler on matrix properties. In more detail, in the glassy zone, the neat polymer has greater E’ values than the composites, while over the transition zone, the E’ of PHB then falls close to the modulus of composites. Melo et al. [37] observed the same behavior in the case of PHB and short carnauba fibers (up to 10% wt.), especially in the case of untreated fibers. The authors concluded that the lower strength of composites compared to the basic matrix was due to the poor interfacial adhesion between the two phases.

The curves of the dissipation factor (tan δ) show a transition (β relaxation) corresponding to a temperature range from −20 to 40 °C for PHB and up to 60 °C for all the other systems. The temperature corresponding to the local maximum of the dissipation factor can be used to determine the glass transition temperature (T_g_). For the pure polymer (PHB), the glass transition temperature is around 23 °C. For the processed polymer (PHB_2), the peak becomes more evident, enlarged, and shifts toward higher temperatures. This variation, due to the extrusion process, is the result of two opposing effects: densification, which reduced polymer chain mobility, and chain scission (due to thermal degradation), which improves macromolecular mobility [55]. The two curves of PHB_2 and PHB/MCC 1.5% overlap, and no significant effect can be observed due to the addition of a 1.5 wt.% filler content. The glass transition temperature is approximately of 28 °C in both cases. A higher amount in cellulose (3 wt.%) leads to a decrement in peak intensity and an increase in the glass transition temperature (~33 °C). This implies a restriction of PHB chain mobility in the presence of fibers [43].

## 4. Conclusions

PHB–cellulose composites at content of 1.5 and 3 wt.% were manufactured in an extruder using a two-fold melt-blending procedure in order to achieve a good dispersion of the filler in the matrix and produce a filament suitable for our 3D-printer.

The process (extrusion + printing) produced some degradation of the polymer that reduced the mechanical properties of pure PHB under both tensile and flexural conditions. The dynamic–mechanical properties of the PHB also changed after processing: the storage modulus (E’) reduced and the glass transition temperature increased. This behavior was interpreted as the result of two opposite mechanisms, a densification and a chain scission, which have contrasting effects on the macromolecular mobility.

Morphological analysis demonstrated a good dispersion of the filler (fibers) within the matrix but a poor interfacial adhesion. The introduction of cellulose into PHB did not alter the crystalline degree and melting temperatures of the polymer but increased its thermal stability as proved by thermogravimetric analysis. Dynamic measurements confirmed that the same weight loss occurred at higher temperatures when the material was loaded with cellulose. Isothermal testing revealed that that the presence of cellulose increased material deterioration in an oxidizing environment, at high temperatures, and throughout a test time of more than 1000 s.

The addition of up to 3% wt. cellulose to processed PHB enhanced its Young modulus, allowing it to achieve the same mechanical properties as virgin polymer. However, similar benefits in flexural properties (both in static and dynamic conditions) were not demonstrated. Even though the addition of cellulose fibers raised the flexural strength and modulus of the processed polymer matrix, the values remained lower than the unprocessed resin. This result was attributed to the poor interfacial adhesion between the two phases in the composite systems.

## Figures and Tables

**Table 1:** Temperatures (T_25_, T_50_, T_75_) in correspondence of specific weight loss percentage (25%, 50%, 75%), and maximum degradation rate (T_max_) for PHB and MB.

	T_25_ (°C)	T_50_ (°C)	T_75_ (°C)	T_max_ (°C)
PHB	290 ± 5	298 ± 4	307 ± 4	296 ± 2
MB	290 ± 4	305 ± 4	320 ± 1	307 ± 3

**Table 2:** DSC data reporting the melting and crystallization temperatures (T_m_ and T_c_), melting and crystallization enthalpies (ΔH_m_ and ΔH_c_), crystallinity degree (X_c_) for PHB and MB during thermal cycle.

	1° Heating			Cooling		2° Heating			
	T_m_ (°C)	ΔH_m_ (J/g)	X_c_ (%)	T_c_ (°C)	ΔH_c_ (J/g)	T_m1_ (°C)	T_m2_ (°C)	ΔH_m_ (J/g)	X_c_ (%)
PHB	172	72.6	50	93	64.4	151	164	74.9	51
MB	172	66.7	54	95	62.1	153	165	64.6	52

**Table 3:** Tensile strength (σ_t_), Young modulus (E_t_), strain at break during tensile test (ε_t_), flexural strength (σ_f_), flexural modulus (E_f_), and strain at break during flexural test (ε_f_).

	σ_t_ MPa	E_t_ MPa	ε_t_ %	σ_f_ MPa	E_f_ MPa	ε_f_ %
PHB	18 ± 2	1650 ± 60	1.5 ± 0.1	29 ± 1	1630 ± 60	2.5 ± 0.1
PHB_2	13.7 ± 0.2	1460 ± 100	1.5 ± 0.1	24 ± 1	1410 ± 30	2.7 ± 0.2
PHB/MCC 1.5%	15 ± 2	1680 ± 60	1.6 ± 0.3	27 ± 1	1520 ± 30	2.7 ± 0.1
PHB/MCC 3%	15 ± 2	1720 ± 40	1.5 ± 0.3	25 ± 2	1400 ± 200	2.6 ± 0.1

## Data Availability

The data presented in this study are available on request from the corresponding author.
